# Rosehip Extract-Loaded Liposomes for Potential Skin Application: Physicochemical Properties of Non- and UV-Irradiated Liposomes

**DOI:** 10.3390/plants12173063

**Published:** 2023-08-25

**Authors:** Aleksandra A. Jovanović, Bojana Balanč, Mina Volić, Ilinka Pećinar, Jelena Živković, Katarina P. Šavikin

**Affiliations:** 1 Institute for the Application of Nuclear Energy INEP, University of Belgrade, Banatska 31b, 11080 Belgrade, Serbia; 2 Innovation Centre of the Faculty of Technology and Metallurgy, University of Belgrade, Karnegijeva 4, 11000 Belgrade, Serbia; bisailovic@tmf.bg.ac.rs (B.B.); mvolic@tmf.bg.ac.rs (M.V.); 3 Faculty of Agriculture, University of Belgrade, Nemanjina 6, 11080 Belgrade, Serbia; ilinka@agrif.bg.ac.rs; 4 Institute for Medicinal Plants Research “Dr Josif Pančić”, Tadeuša Košćuška 1, 11000 Belgrade, Serbia; jzivkovic@mocbilja.rs (J.Ž.); ksavikin@mocbilja.rs (K.P.Š.)

**Keywords:** encapsulation, liposomes, proliposome method, Raman spectroscopy, *Rosa canina*, UV irradiation

## Abstract

In the present study, rosehip (*Rosa canina* L.) extract was successfully encapsulated in phospholipid liposomes using a single-step procedure named the proliposome method. Part of the obtained liposomes was subjected to UV irradiation and non-treated (native) and UV-irradiated liposomes were further characterized in terms of encapsulation efficiency, chemical composition (HPLC analysis), antioxidant capacity, particle size, PDI, zeta potential, conductivity, mobility, and antioxidant capacity. Raman spectroscopy as well as DSC analysis were applied to evaluate the influence of UV irradiation on the physicochemical properties of liposomes. The encapsulation efficiency of extract-loaded liposomes was higher than 90%; the average size was 251.5 nm; the zeta potential was −22.4 mV; and the conductivity was found to be 0.007 mS/cm. UV irradiation did not cause a change in the mentioned parameters. In addition, irradiation did not affect the antioxidant potential of the liposome–extract system. Raman spectroscopy indicated that the extract was completely covered by the lipid membrane during liposome entrapment, and the peroxidation process was minimized by the presence of rosehip extract in liposomes. These results may guide the potential application of rosehip extract-loaded liposomes in the food, pharmaceutical, or cosmetic industries, particularly when liposomal sterilization is needed.

## 1. Introduction

*Rosa canina* L. (Rosaceae) is frequently used in traditional medicine, due to its diuretic, anti-inflammatory, anti-allergic, antioxidant, and analgesic properties. Dried rosehips (*Rosae pseudo-fructus*) are most often used in phytotherapy and are included in European Pharmacopoeia. Rosehip is known for its preventive and curative activities against a wide range of renal, inflammatory, gout, and gastric diseases [1]. Several studies have shown that rosehip and its constituents possess antioxidant, antimicrobial, anti-inflammatory, analgesic, mild laxative, diuretic, and anti-cancerogenic properties and have a positive effect on dermatoses, ulcers, and other skin diseases [2,3,4,5]. Therefore, rosehip extracts are becoming popular in the pharmaceutical, cosmetic, and agro-food industries. *R. canina* fruits and extracts are rich in various bioactive compounds, including polyphenols, carotenoids, sugars, ascorbic acid, tocopherol, mineral elements, pectins, amino acids, and fatty acids [6,7]. However, the functionality, stability, and bioavailability of active compounds, particularly polyphenols, to which some biological effects are linked, mostly depend on several factors such as light, pH, temperature, irradiation, free radicals, and storage conditions. Thus, one of the effective approaches to preserve the beneficial properties of polyphenols is the application of encapsulation techniques [8]. The main advantages of encapsulation are the increment stability of bioactive compounds during storage, protection from harmful environmental conditions and the effects of components present in the skin or gastrointestinal tract, increase in bioavailability, modification in delivery of active compounds, and extension of product shelf life [9]. One of the many encapsulation methods is liposomal entrapment, which presents a simple, precise, efficient, and economical technique. The mentioned encapsulation technology is suitable for the production of nano-sized particles that are favored from the viewpoint of textural and sensorial characteristics of food, cosmetic, and pharmaceutical products [9,10]. Liposomes, as nanoparticles, are widely used as a carrier for the delivery of drugs, antioxidants, proteins, enzymes, polyphenols, vitamins, flavors, and aromas, due to their non-toxicity, biodegradability, and ability to encapsulate hydrophilic, amphiphilic, and lipophilic components. They also can provide a controlled release of bioactive compounds and protection from light, oxygen, UV irradiation, free radicals, enzymes, and changes in pH values. Additionally, liposomes ensure a higher bioavailability of various compounds, including drugs, proteins, nutraceuticals, and polyphenols, due to their chemical structure being similar to epithelial cells [11]. It is important to emphasize that an adequate characterization of liposomes is essential, with the aim being to ensure that these vesicles encompass the required and expected properties for their specific application. Concerning potential implementation in food, cosmetic, or pharmaceutical industries (bandages for wounds and burns), liposomal sterilization remains a real challenge because of the carriers’ particular sensitivity and physicochemical alterations [11]. UV irradiation, as a sterilization technique, provides rapid and successful inactivation of chemicals or corrosive compounds without the production of carcinogenic substances and is highly effective against a wide range of bacterial strains [12]. However, UV irradiation can have a limited penetration range. Nevertheless, Tapia-Guerrero et al. [13] study has shown that UV sterilization, as a terminal method, can be scaled to industrial levels and kill viruses, bacteria, yeast, and fungi providing a safe and effective principle of sterilization in the food and pharmaceutical industries (where UV lamps are used for irradiation) and extending shelf life and nutritional value. Additionally, irradiation with shortwave ultraviolet light, such as UV-C (electromagnetic radiation with germicidal properties at 250–270 nm), possesses various technological advantages due to minimal energy use, low maintenance, installation costs, and preservation of the products without some undesirable impact of heat treatments [12]. Specifically, UV-C irradiation represents an effective technique for inactivating pathogenic and spoilage microorganisms in various products in the medical and food areas due to the formation of lesions in DNA within microorganisms (breaking molecular bonds, creating new bonds between nucleotides, and producing thymine dimers), inhibition of cellular enzyme activity, and destruction of cytoplasmic membrane integrity [12,13,14]. UV-C irradiation in the pharmaceutical industry has an important role in microbial contaminants disinfection, breaking down of ozone, dechlorination of water, as well as total organic carbon reduction. Also, the influence of UV light irradiation on pharmaceutical or cosmetic skin formulations’ ingredients (such as liposomes) should be investigated as a simulation of sunlight irradiation [15].

In the present study, phospholipid liposomes, as the carriers for rosehip extract, were developed via the proliposome method. To obtain a high-quality product, liposomes were characterized via the investigation of encapsulation efficiency (EE), chemical composition, antioxidant potential, particle size, polydispersity index (PDI), zeta potential, conductivity, mobility, and thermal stability (differential scanning calorimetry, DSC). Raman spectroscopy was also applied to evaluate the influence of UV irradiation on the chemical properties of liposomes. To the best of our knowledge, there was no investigation of rosehip extract-loaded liposomes intended for skin use, as well as the influence of UV irradiation on the physicochemical properties of the obtained liposomes.

## 2. Results

In the present study, liposomes with rosehip extract were developed and characterized in terms of encapsulation efficiency, antioxidant capacity, particle size, PDI, zeta potential, conductivity, mobility, and thermotropic properties. Stability after the UV irradiation of liposomes and Raman spectroscopy analysis were assessed as well. The chemical composition of *R. canina* extract and extract-loaded liposomes was also investigated.

### 2.1. Encapsulation Efficiency

Since encapsulation efficiency represents one of the most important parameters characterized by the encapsulation process [16], the encapsulation efficiency of rosehip polyphenols within liposomes is determined and shown in Table 1. Considering that food, pharmaceutical, and cosmetic industries use UV irradiation for sterilization purposes, the encapsulation efficiency was determined immediately after the liposomal preparation and after UV irradiation.

The encapsulation efficiency of rosehip extract in liposomes was 90.8 ± 0.4% (Table 1). Further, UV irradiation did not cause a decrease in the encapsulation efficiency of rosehip polyphenols in liposomes, 91.0 ± 0.8% (Table 1). Specifically, UV irradiation did not result in a leakage of encapsulated compounds of the extract into surrounding water in liposomal suspension. The TPC of the rosehip extract was 9.64 ± 0.98 mg GAE/mL, while in the UV-irradiated extract, TPC was 9.54 ± 0.30 mg GAE/mL. Specifically, no statistically significant difference in TPC between native and UV-irradiated extracts was found.

### 2.2. HPLC Analysis of the Extract and Extract-Loaded Liposomes

The quantification of individual compounds in *R. canina* extract and extract-loaded liposomes was carried out using the HPLC method, and the results are shown in Table 2.

The main compound in the extract was isoquercetin, followed by rutin, while the contents of hyperoside, quercitrin, and chlorogenic acid were significantly lower. In the liposomal sample, isoquercetin and rutin were the most dominant components. Nevertheless, the content of chlorogenic acid in the liposomes was higher in comparison to hyperoside and quercitrin, which was different than in the case of the extract. As can be seen from Table 2, UV irradiation caused a decrease in the content of all mentioned individual compounds in extract-loaded liposomes.

### 2.3. Physical Properties of Rosehip Extract-Loaded Liposomes

Physical properties of empty and rosehip extract-loaded liposomes (particle size, PDI, zeta potential, conductivity, and mobility) were investigated before and after UV irradiation; the results are presented in Table 1.

#### 2.3.1. Size of Liposomes

Particle size, besides size distribution and zeta potential, represents fundamental parameters characterizing colloidal dispersion [16]. As can be seen from Table 1, all populations of liposomes have a size higher than 100 nm which indicates at least a double-bilayer structure. Additionally, UV irradiation did not cause a change in the liposomal particles size (Table 1). The liposomal particles with encapsulated extract had statistically significantly smaller diameters (~250 nm) than empty liposomes (~360 nm) in both cases (non-treated and UV-irradiated samples).

#### 2.3.2. PDI of Liposomes

PDI value, as a dimensionless parameter, is derived from cumulant analysis and it stands for the width of Gaussian size distribution [16]. Regarding the PDI of liposomal suspension, extract-loaded liposomes had higher PDI (~0.440) in comparison to empty liposomes (~0.380), while UV irradiation did not cause changes in PDIs (Table 1).

#### 2.3.3. Zeta Potential of Liposomes

Zeta potential, as an indicator of the overall stability of the liposomal vesicles, was also determined for unloaded and extract-loaded liposomes (non-treated and UV-irradiated samples, Table 1). All samples possessed a negative value of zeta potential in the absence or presence of the encapsulated rosehip extract (from −21.3 to −22.4 mV); the zeta potential of the pure extract was −0.59 ± 0.02 mV. The encapsulation of the extract, as well as UV irradiation, did not cause a statistically significant change in the zeta potential of liposomes (Table 1).

#### 2.3.4. Conductivity of Liposomes

The measurement of liposome conductivity represents one of the aspects of liposome characterization and possibly stability assessment. According to the PCS measurements, the conductivity of unloaded liposomes was slightly lower (0.004 ± 0.000 mS/cm) than the conductivity of rosehip extract-loaded liposomes (0.007 ± 0.001 mS/cm), whereas UV irradiation did not affect the mentioned parameter (Table 1). The conductivity of pure rosehip extract was 0.046 ± 0.002 mS/cm.

#### 2.3.5. Mobility of Liposomes

Mixtures of liposomes and other compounds, such are drugs, extracts, or polymers, represent a flexible formulation platform exhibiting different particle mobility depending on the characteristics of the surface of the liposome. Therefore, it is possible to modulate the target place for the release of encapsulated compounds and thus its release kinetics [17]. However, the release from liposomes depends on the physicochemical characteristics of the active compounds and liposomes, as well as of the surrounding environment [18]. Nevertheless, the mobility of liposomes is a key parameter for the delivery of active compounds. This mobility property is correlated to the interactions between liposomes and encapsulated compounds, as well as to the microstructure of the system [17]. Empty liposomes exhibited a mobility of ~−2.5 µmcm/Vs, whereas liposomes with rosehip extract had a mobility of ~−1.9 µmcm/Vs (Table 1). Additionally, UV irradiation did not cause significant changes in liposome mobility (Table 1).

### 2.4. Antioxidant Capacity of Rosehip Extract and Liposomes

**Figure 1:**
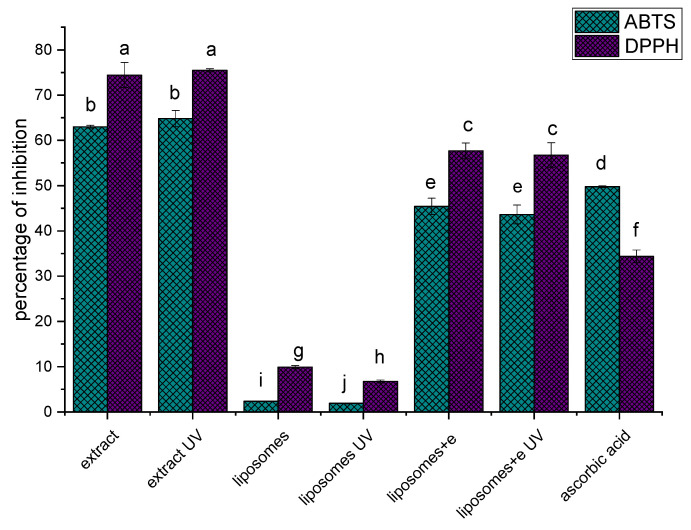
Antioxidant capacity of rosehip extract and of empty and extract-loaded liposomes before and after UV irradiation, and ascorbic acid (as a control, at a concentration of 0.2 mg/mL) determined in ABTS and DPPH assays; different letters (a–j) indicate that there was a statistically significant difference based on Duncan’s *post hoc* test at *p* < 0.05 level, n = 3, mean value ± standard deviation.

Various antioxidants are used to increase the oxidation stability of phospholipids, including α-tocopherol and butyl hydroxyl toluene. However, liposomes can be used as a carrier for both liposoluble and hydrosoluble antioxidants to enhance their bioavailability and at the same time, liposomes can be protected from oxidation [19]. Thus, the antioxidant capacity of rosehip extract and of unloaded and extract-loaded liposomes (before and after UV irradiation) was determined via ABTS and DPPH radical scavenging activity assays, and the results are presented in Figure 1.

As can be seen from Figure 1, pure rosehip extract (diluted to concentration as in liposomes) inhibited 63.0 ± 0.3% of ABTS radicals and 74.4 ± 2.8% of DPPH radicals before UV irradiation, and 64.8 ± 1.8% of ABTS radicals and 75.5 ± 0.4% of DPPH radicals after UV irradiation. It can be noticed that UV irradiation did not cause statistically significant changes in the antioxidant potential of pure extract. On the other hand, the antioxidant activity of unloaded liposomes, which originated from synthetic antioxidants added to Phospholipon, was 2.4 ± 0.1% of ABTS radical inhibition and 9.9 ± 0.3% of DPPH radical inhibition before irradiation, and 1.9 ± 0.1% of ABTS radical inhibition and 6.7 ± 0.3% of DPPH radical inhibition after irradiation. Therefore, in plain liposomes, UV irradiation caused a decrease in antioxidant potential. Liposomes with rosehip extract neutralized 45.4 ± 1.8% of ABTS radicals and 57.7 ± 1.7% of DPPH radicals before UV irradiation, and 43.6 ± 2.1% of ABTS radicals and 56.7 ± 2.7% of DPPH radicals after UV irradiation. As in the case of pure extract, irradiation did not affect the antioxidant potential of the liposomes–extract system. Ascorbic acid (0.2 mg/mL) neutralized 49.7 ± 0.2% of ABTS radicals and 34.4 ± 1.4% of DPPH radicals.

### 2.5. Raman Spectroscopy

Raman spectroscopy was applied with the aim to examine the presence of different interactions among the rosehip extract, Phospholipon, liposome carrier with and without loaded extract, and its UV-treated counterparts.

**Figure 2:**
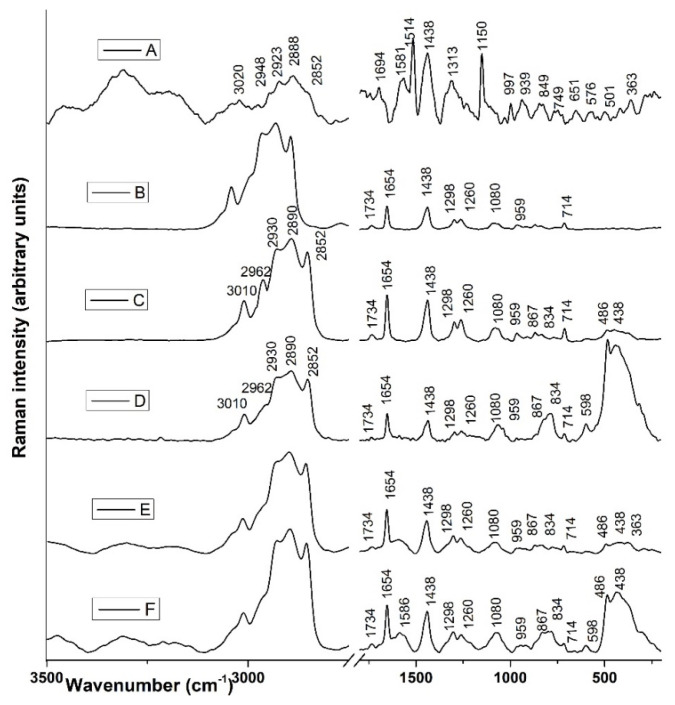
Averages of normalized Raman spectra of rosehip extract (**A**), Phospholipon (**B**), liposomes (**C**), liposomes treated with UV irradiation (**D**), liposomes with extract (**E**), and liposomes with extract treated with UV irradiation (**F**), in the spectral range from 250 to 3500 cm^−1^.

The Raman spectrum of rosehip extract (Figure 2A) exhibits the most dominant bands indicated on carotenoids as the major and essential constituents of rosehip hypanthium cells. The bands observed at 1514, 1150, and 997 cm^−1^ originate from the C=C, C–C stretching vibrations of the polyene backbone, and methyl groups (C–CH_3_) attached to the conjugated polyene skeletons, respectively [20,21,22]. The medium intensity bands (Figure 2A) at 1694 cm^−1^ indicated the amide I band, predominantly β-sheet [23], and bands at 1581 and 1438 cm^−1^ could indicate carotenoids and lipids, respectively [20,22,24], while the band at 1313 cm^−1^ could be attributed to CH_2_ bending vibration of cellulose or polygalacturonic (pectic) acid [23]. Commonly, the bands ranging from 350 to 940 cm^−1^ probably refer to glycosidic links or ring stretches [22]. A higher intensity broad in the rosehip extract spectrum ranging at 2852 and 2888 cm^−1^ originates from CH_2_ and CH_3_ symmetric stretching vibrations, while bands at 2923, 2948, and 3020 cm^−1^ could indicate asymmetric CH_2_ and =C–H vibrations of phenyl moieties [21,22].

The Raman features of the Phospholipon (Ph) and liposome (L) spectra (Figure 2B,C) correspond to phospholipid head-group C–N symmetric stretching of choline at 714 cm^−1^, then C–C and C–H modes from the lipid acyl chains from phospholipids, including the band at 1080 cm^−1^ which can be assigned to the skeletal stretching of the C–C vibrations, 1298 cm^−1^ (in-plane CH_2_ twisting mode) and 1438 cm^−1^ (CH_2_ scissoring mode) [21,25,26,27,28,29,30]. Phospholipon also exhibited additional Raman bands at 1260 and 1654 cm^−1^ that could be assigned to *cis* =C–H and C=C stretching vibration in the oleoyl chain [21,28,30] and the ester carbonyl stretch at 1734 cm^−1^ [28].

In the lipid membrane, lipid chains are fluid compared to hydrophilic heads and vibration coming from methylene modes could be shifted to the wavenumbers from 2850 to 3015 cm^−1^. In Phospholipon and liposomes (Figure 2B,C), the symmetric and antisymmetric CH_2_ stretching vibrations perform bands at 2852 and 2890 cm^−1^, respectively [28]. The following bands at 2930 and 2962 cm^−1^ arose from symmetric, antisymmetric methyl stretches of fatty acids chains, while the band at 3010 cm^−1^ could indicate vinylic C–H vibrations that originated from the oleoyl chain [28]. Larsson and Rand [31] indicated that the C–H stretching region from 2850 to 2890 cm^−1^ could explain hydrocarbon chain bands in lipids due to the interaction between lipid and water phases [21]. 

On the differences between Phospholipon and liposomes (Figure 2B,C), Raman spectra could indicate a slightly higher intensity of the band in the lipid fingerprint region from 1660–430 cm^−1^. The changes also include the appearance of the bands which could indicate the presence of palmitic and stearic acids esters [26], such as bands at 438 and 486 cm^−1^. These bands have strongly higher intensities in the samples treated with UV irradiation, as well as medium intensity bands located at 834 and 867 cm^−1^ (Figure 2D,F), as the most characteristic features of stearic acid [26].

The Raman spectra of liposomes with encapsulated rosehip extract (Figure 2E) mostly resembled plain liposomes (Figure 2C).

### 2.6. Thermal Properties of Rosehip Extract and Liposomes

**Figure 3:**
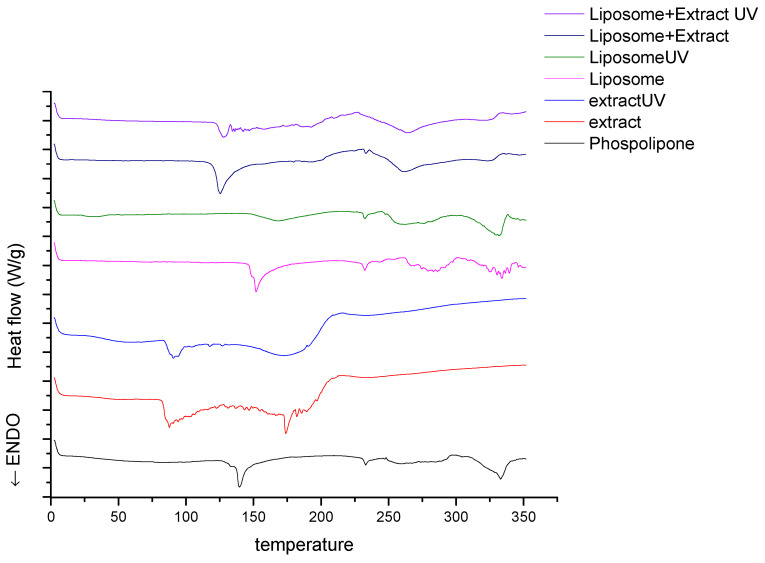
Differential scanning calorimetry curves of rosehip extract-loaded liposomes, unloaded liposomes, pure extract (UV-irradiated and non-treated samples), and pure Phospholipon.

DSC is a tool to study the thermal behavior of lipid membranes and lipid drug delivery systems, such are liposomes, by measuring thermodynamic parameters (T_m_, gel-to-liquid crystalline phase transition temperature and ΔH, enthalpy), which influence the stability of liposomes. Since the stability of nanoparticles with biologically active compounds is of great importance, thermal analysis can provide detailed information on their storage stability and also stability during the development processes [32]. Hence, DSC analysis of pure rosehip extract and of unloaded and extract-loaded liposomes (non-treated and UV-irradiated samples) was performed. The results of the DSC analysis are represented as curves (Figure 3), as well as T_m_ and ΔH values (Table 3).

Figure 3 presents DSC curves of pure Phospholipon, plain liposomes, extract, liposomes containing the extract, and the same samples treated with UV irradiation, while the changes in enthalpy are listed in Table 3. Curve derived from Phospholipon displays a sharp endothermic peak at 138.7 °C (−22.7 J/g). The extract showed one endothermic peak around 87.8 °C (−38.5 J/g). The thermal decomposition occurs in the temperature range between 120 °C and 190 °C where several endothermic peaks were observed, as well as one intensive peak around 174.4 °C (−7.5 J/g). In the DSC curve of plain liposomes, a sharp endothermic peak is noticed at 152.4 °C (−30.5 J/g) and several thermal events occurred starting from 230 °C implying further degradation. Further, when observing the DSC curve, which corresponds to liposomes with encapsulated extract, the change in peak position is evident. The main endothermic peak is shifted to 125.6 °C with a minor change in enthalpy (−32.8 J/g). In the DSC spectra of the UV-treated analogs, the peaks are less intense and wide, with decreased values of enthalpy change.

## 3. Discussion

According to the literature, hydrophobic and hydrophilic bioactive compounds can be encapsulated into lipid vesicles with encapsulation efficiency between 80 and 90%. However, the encapsulation efficiency of polyphenols from plant extract is usually lower in liposomes, 61–72% for hibiscus (*Hibiscus sabdariffa*) extract [33], 40–66% for black carrot (*Daucus carota*) extract [19], 25–69% for elderberry (*Sambucus nigra*) extract [16], and 25–58% for olive pomace (*Olea europaea*) extract, even with the application of a supercritical assisted process that enhances encapsulation efficiency [34]. Nevertheless, the encapsulation efficiency of polyphenols in liposomes containing grape seed extract was higher and amounted to 93%, but it was performed by using high-pressure homogenization [35]. In our case, a fairly high encapsulation efficiency of rosehip polyphenols (which, according to the literature, include 4-hydroxybenzoic, vanillic and syringic acids, and methyl ester *p*-coumaric acid [3]) was obtained by applying a simple and economic proliposome method. The proliposome method used in our study can be applied for the encapsulation of both hydrophilic and lipophilic bioactive compounds from various plant extracts due to the specific structure and composition of the liposomal particles which can entrap target compounds inside (in water surrounding, for hydrophilic) or between phospholipids tails (for lipophilic).

Previous studies reported that the particle size of liposomes was highly dependent on the liposomal composition, as well as on the material that was encapsulated within lipid vesicles [33,36]. However, in the case of green tea or black carrot extract-loaded liposomes, the particle size was the same for loaded and unloaded liposomes [19,36]. Our results are in agreement with other literature data, such as those published by Lopez-Pinto et al. [37], who have reported that the presence of ethanol residues influenced the particle size of liposomes, thus causing a change in the net charge and providing steric stabilization. Since the encapsulated rosehip extract was 70% ethanolic, it can be the reason for the decrease in liposome size. Also, the encapsulation of polyphenols may reduce the number of lipid compounds involved in the formation of the liposome membrane [38,39], consequently creating smaller particles. The study by Temprana et al. [40] has shown that changes induced by UV irradiation are sufficient to increase the size stability of liposomal populations. In contrast to our results, Toopkanloo et al. [41] reported that the vesicle size of the liposomes increased due to exposure to UV irradiation and due to their photochemical destruction via absorption of photon energy and a drastic change in the liposome bilayer conformation. However, in the case of the mentioned study, liposomes contained cholesterol and Tween 80 apart from soy phospholipids, which made the liposomal bilayer more fluid and permeable and, consequently, more susceptible to UV-induced disturbing of the order and lipid packing of the membrane. On the other hand, the liposomes with rosehip extract contained only phospholipids (without the addition of sterols) that formed a more rigid membrane bilayer [42].

The obtained values for PDI are in agreement with the literature, wherein the PDI of soybean lecithin liposomes with elderberry extract was ~0.490 [16]. Specifically, the lipid concentration and composition, liposome preparation technique, as well as the duration of mixing affect the dispersity of liposomal suspension [42,43]. Also, Trucillo et al. [34] reported that at higher bioactive compound loading of liposomes, larger dispersions were obtained, and PDIs increased with the increasing content of olive pomace extract within the liposomes. Ardani et al. [44] have reported that PDI values over 0.4 indicated the existence of a moderately dispersed distribution. According to the literature data, a single phospholipid, such as 1,2-dipalmitoyl-*sn*-glycero-3-phosphocholine, provides better uniformity (PDI of ~0.1) compared to a mixture of phospholipids (such as Phospholipon, a commercial phospholipid mixture used in the present study). Specifically, a single phospholipid eliminates the imperfect packing that can occur in the case of various hydrophobic fatty acyl chain lengths, head groups, and degrees of saturation present in the mixture [42]. Additionally, higher liposomes (vesicle size over 1000 nm) possessed lower PDIs in comparison to a smaller liposomal population (particle size from 100 to 400 nm) [42]. Since PDI represents a measure of the vesicle size distribution, it was expected that UV light irradiation did not affect PDI either, as in the case of the size of the liposome’s population.

The results obtained for zeta potential are in agreement with our previous studies where the encapsulation of gentisic acid and wild thyme ethanol extract did not cause a change in the zeta potential of liposomes in comparison to plain liposomes [45,46]. Moreover, the encapsulation of polyphenols from green tea extract did not provoke significant change in the zeta potential of the liposomal membrane as well [36]. In the case of olive pomace extract-loaded liposomes, zeta potential and overall stability of lipid vesicles are not affected by the increase in the quantity of entrapped compounds [34]. Although UV exposure can cause comprehensive zeta potential change in the liposomal suspension or even reversal from negative to positive [47], it was not the case with rosehip extract-loaded liposomes. It can be explained by the fact that UV light does not usually have to induce any large-scale reorganization of the phospholipid bilayer or disruption of membrane integrity, nor leakage of entrapped molecules (which was proven via the measurement of EE and conductivity, Table 1) [47].

The obtained results of conductivity values were expected since Froude and Zhu [48] indicated that positively charged colloids could stabilize anionic liposomes more effectively than the negatively charged ones, while the addition of negative colloids appears equivalent to adding salts or macroions to effectively vary the medium conductivity, rather than liposome surfaces. Also, the conductivity of the inner medium of liposomes can be modified by encapsulated compounds. An additional explanation for the higher conductivity of the liposomes with rosehip extract should be in decreased lipid concentration within the liposomal bilayer since there is a competition between lipids and extract compounds for space in the membrane [38,39]. Specifically, lower amounts of ions are removed as the liposome capture volume decreases with decreasing lipid concentration [39]. As a consequence, the conductivity of the liposome dispersions increases as the lipid concentration decreases. In the case of higher lipid concentration, when ions are inside the liposomal particles, their mobility is reduced, and their contribution to conductivity is no longer apparent. Greater lipid concentrations = higher capture volume = the effective removal of ions from the liposome dispersions = the reduction in conductivity. The conductivity of the liposome dispersion was influenced by the exposed charge of the phospholipids as well. Small liposomes most notably affected conductivity, due to greater surface area which exposes a higher percentage of phospholipid head groups [39]. Thus, the measured conductivity of the liposomes with rosehip extract was greater (smaller particles than of unloaded liposomes). Bordi et al. [39] also demonstrated a clear correlation between conductivity and volume of liposome entrapment. Since there was no statistically significant difference in conductivity values between non-treated and UV-irradiated extract-loaded liposomes, it can be concluded that UV light did not cause leakage of encapsulated compounds.

The obtained varieties in the mobility of different liposomes are expected since mobility is a function of vesicle size, zeta potential, and bilayer composition [49]. Although vesicles with lower charge should correspondingly have lower mobility, it is not valid for all systems. Thus, Pysher and Hayes [50] demonstrated that the population of liposomes with the highest charge exhibited the lowest mobility. In our study, liposomes with the highest charge (unloaded samples) also showed statistically significant lower mobility, but the difference between charges was not statistically significant. It can be explained through consideration of the unique characteristics of liposome populations. Specifically, some liposomal membranes are rigid and non-deformable, while others are fluid, squishy, and capable of undergoing a variety of shape deformations in response to externally applied pressure and shear forces, which depend on bilayer composition and encapsulated compounds [42,50]. Since any changes in the liposomes’ mobility were attributed to the mechanical rigidity, or the ability of the liposomes to deform [50], it can be concluded that unloaded liposomes were softer and less rigid than liposomes with a rosehip extract, which exhibited lower mobility. Furthermore, flavonoid compounds can be adsorbed at the surface of the mentioned liposomes with extract, thus reducing their mobility [51]. However, the relatively minor change in mobility between unloaded and loaded liposomes does not account for the significant changes observed (approximately 20%). It was expected that UV irradiation did not cause significant changes in liposome mobility due to the correlation between mobility and zeta potential or particle size [49], as parameters that did not change after UV exposure.

Regarding the results from antioxidant assays, it can be concluded that antioxidant compounds from rosehip extract in a given concentration protect liposomes more effectively than added synthetic antioxidants. The obtained results of antioxidant activity are correlated to the polyphenol content in pure extract and liposomes (explained in Section 2.1). The correlation between the antioxidant capacity of extracts and polyphenol content was established in several studies [52,53,54]. In all samples, DPPH radical scavenging activity was statistically significantly higher than the antioxidant capacity shown in the ABTS assay. Taneva et al. [54] also demonstrated that rosehip extract more effectively neutralized DPPH radicals in comparison to ABTS radicals. The reason for different radical scavenging potentials can lie in the fact that the antioxidant activity of rosehip extract depends not only on the polyphenol content but also on ascorbic acid and tannins [54].

Since the Raman spectra of liposomes with encapsulated rosehip extract (Figure 2E) mostly resembled empty liposomes (Figure 2C), it may indicate that the extract is completely covered by the bilayer membrane during liposome entrapment (its signature spectrum was hidden). Similar findings were published by Hosseini et al. [55] and Šeremet et al. [56]. Further, the Raman spectra of UV-treated liposomes with encapsulated rosehip extract (Figure 2E), compared with the other spectrums in Figure 2, indicated the appearance of the band at 1586 cm^−1^, which could originate from the polyene chain [24] from rosehip extract, thus implying changes in carotenoid compounds. Specifically, Nakata et al. [29] explained that the C=C bonds could undergo oxidation or polymerization or any kind of structural changes, or the concentration of molecules nearby could induce the appearance of new bands. 

The possible structural changes in the system provided by liposome and plant extract treated with UV irradiation could indicate the increasing intensity of the broad bands in the regions from 430 to 490 cm^−1^ and from 830 to 870 cm^−1^, which suggests that UV has an impact on the chemical concentration of the fatty acids or its esters (phosphatidylcholine, the main ingredient of Phospholipon, belongs to ester phospholipids). An increase in band intensity at 438, 486, 834, and 867 cm^−1^ could indicate a peroxidation of phosphatidylcholine irradiated by UV light (Figure 2C,D). The peroxidation process is minimized by the presence of rosehip extract loaded in liposomes (Figure 2D,F) as a result of the antioxidant properties of its polyphenolic compounds.

A sharp endothermic peak from Phospholipon curve is probably linked with phase transition (gel to liquid). Maiti et al. [57] commented that melting of the non-polar hydrocarbon phospholipid tails during this phase may yield a sharp peak, while endothermic peaks at 233.6 °C and 333.9 °C were observed due to possible degradation [58]. One endothermic peak from the extract curve is probably linked to the loss of volatile compounds, such as ethanol. The presence of multiple peaks from the extract curve can be ascribed to the various compounds of the extract such as secondary metabolites, mainly polyphenols. Martin Ramos et al. [59] investigated the oil extract of *Rosa rubiginosa*, and they found that the thermal effects around 175 °C can be ascribed to a transformation of pentahydrate into an anhydride upon temperature increase or to lycopene since the melting of this red pigment occurs around 173 °C. In the DSC curve of plain liposomes, a sharp endothermic peak and several thermal events were noticed, which is in accordance with other authors [60]. Further, when observing the DSC curve which corresponds to liposomes with encapsulated extract, the change in peak position is evident. This result was expected since the other authors also reported this phenomenon when polyphenolic compounds or flavonoids are incorporated in liposomes [57,61,62]. Specifically, the molecules of active compounds such as flavonoids act as spacers, triggering a destabilization of the lipids with a decrease in the temperature of the gel to the liquid–crystal phase transition.

Since the peaks of UV-treated analogs are less intense and wide, with decreased values of enthalpy change, it indicates that UV irradiation changes the shape of the DSC curves, and it is also confirmed by other authors who concluded that the broadening of the DSC curves after UV irradiation can be triggered by the alteration of crystallites’ size and their distribution [63]. In addition, in the spectra of UV-treated extract, a characteristic peak around 173 °C, which is typical for carotenoids, is barely noticeable (the enthalpy change is −2.2 J/g), implying the degradation of these compounds during irradiation. Carotenoids are highly susceptible to oxidation or isomerization since they have conjugated double bonds in the molecule, and thus, exposure to UV light led to damage in the molecule [64].

## 4. Materials and Methods

### 4.1. Plant Material and Reagents

Dried rosehips without seeds (*Rosae pseudo-fructus*) were obtained from the production sector of the Institute for Medicinal Plants Research ”Dr Josif Pančić”, Serbia (voucher specimen: UBFA1353A-J). They were collected in Veliko Bonjince, near the town of Leskovac, Southern Serbia, at the end of September 2022. The following reagents were used: ethanol and sodium carbonate (Fisher Scientific, Loughborough, UK), Folin–Ciocalteu reagent and gallic acid (Merck, Darmstadt, Germany), Phospholipon 90 G (unsaturated diacyl-phosphatidylcholine) (Lipoid GmbH, Ludwigshafen am Rhein, Germany), potassium persulfate (Centrohem, Stara Pazova, Serbia), orthophosphoric acid, acetonitrile, 2,2′-azino-bis(3-ethylbenzothiazoline-6-sulphonic acid) or ABTS, and 2,2-diphenyl-1-picrylhydrazyl or DPPH were from Sigma-Aldrich (Burlington, MA, USA).

### 4.2. Extract Preparation

Rosehip extract was obtained using percolation at ambient temperature (25 °C). Grounded and sieved dried rosehip shells were extracted using 70% ethanol in a ratio of 1:2. In our preliminary study, we investigated the influence of different extraction mediums (water, water–ethanol mixture, as well as natural deep eutectic solvents, NaDESs) on the polyphenol content of rosehip extracts. Water–ethanol mixture gave statistically significantly higher polyphenol yield in comparison to water parallel. Thus, 70% ethanol extract was chosen for encapsulation into liposomal particles. In our previous study, total polyphenol content was also measured after ultrasound-assisted extraction of rosehips and amounted to 3.86 ± 0.12 mg gallic acid equivalents (GAE)/g of plant material [65]. Additionally, NaDESs significantly improved polyphenol recovery in comparison to water as well (6.4–10.4 mg GAE/g) [66]. However, because of the very high values of viscosity of NaDES extracts, it is not possible to encapsulate them within liposomes with satisfying encapsulation efficiency.

### 4.3. Liposomes Preparation

Rosehip extract-loaded liposomes were prepared using the proliposome method [43]. Specifically, Phospholipon (1 g) and 70% ethanol rosehip extract (1 mL, solid-to-solvent ratio 1:2) were stirred at 50 °C. After cooling to room temperature, ultrapure water (20 mL) was added in small portions; the emulsion was stirred at 800 rpm for 1 h. Subsequently, the liposomes were sonicated using an ultrasound bath with ultrasonic peak power of 640 W, nominal ultrasonic power of 160 W, and ultrasonic frequency of 35 kHz (SONOREX, Bandelin, Germany) for 30 min. Additionally, liposomes without the extract were prepared as a control.

### 4.4. Lyophilization

Lyophilized samples (rosehip extract, empty and extract-loaded liposomes) were prepared for DSC analysis; ethanol (from the extract) was removed at 50 mbar and 40 °C, using a Heizbad Hei-VAP rotary evaporator (Heidolph, Schwabach, Germany). Further, the samples were frozen in a freezer, LAB11/EL19LT (Elcold, Hobro, Denmark), at −80 °C for 1 h and lyophilized in Beta 2-8 LD plus freeze-dryer (Christ, Osterode am Harz, Germany) at −75 °C and 0.011 mbar for 24 h.

### 4.5. Determination of Total Polyphenol Content and Encapsulation Efficiency

Total polyphenol content (TPC) of native and UV-irradiated extracts and in liposomal supernatant was determined spectrophotometrically at 765 nm using the modified Folin–Ciocalteu method [67]. The results are expressed as milligrams of gallic acid equivalents per milliliter of liquid extract (mg GAE/mL).

Encapsulation efficiency (EE) was determined using the indirect method. EE was calculated as shown in Equation (1):

EE [%] = (TPC_i_ − TPC_sup_)/TPC_i_ × 100 (1)

where TPC_i_ is the initial content of total polyphenols presented in rosehip extract used for the preparation of liposomes, and TPC_sup_ is the content of total polyphenols determined in the supernatant.

The free extract was removed from liposome dispersions by centrifugation at 17,500 rpm and 4 °C for 45 min in Thermo Scientific Sorval WX Ultra series ultracentrifuge (ThermoScientific, Waltham, MA, USA).

### 4.6. HPLC Analysis

Analyses were carried out on an Agilent 1260 RR HPLC instrument (Agilent, Waldbronn, Germany) equipped with a diode-array detector working in the range of 190–550 nm. The samples were separated using a reverse-phase Zorbax SB-C18 (Agilent) analytical column (150 mm × 4.6 mm i.d.; 5 μm particle size). Mobile phase A was a 1% *v*/*v* solution of orthophosphoric acid in water, while mobile phase B was acetonitrile. Gradient elution was performed according to the following scheme: 0–2.6 min, 90–85% A; 2.6–8 min, 85% A; 8–10.8 min, 85–80% A; 10.8–18 min, 80% A; 18–23 min, 80–70% A; 23–25 min, 70–50% A; 25–27 min, 50–20% A; 27–29 min, 20–10% A; 29–34, 0% A. Detection wavelengths were set at 260, 280, 320, and 360 nm, and the flow rate was 1 mL/min. The injection volume was 8 μL, and the column temperature was maintained at 40 °C. Identification of the compounds was achieved by comparing their UV spectra and retention time with those from authentic substances. The amounts of the compounds were calculated using calibration curves. The results are presented as micrograms per gram of dry weight (μg/g dw) for extract-loaded liposomes or as micrograms per milliliter (μg/mL) for extract.

### 4.7. Determination of the Antioxidant Potential of the Extract and Liposomes

The antioxidant capacity of rosehip extract, plain and extract-loaded liposomes (native and UV irradiated) was analyzed according to the previously published methods, ABTS and DPPH assays [53]. Extract-loaded liposomes were centrifuged before antioxidant assays to eliminate the antioxidant influence of non-encapsulated extract. The concentration of pure extract used for the antioxidant assays was 0.5 g/mL (diluted to concentration as in liposomes).

#### 4.7.1. ABTS Assay

The absorbance of ABTS radicals (2 mL) and the samples (20 µL) was spectrophotometrically measured at 734 nm, and calculated as % of inhibition = (A_0_ − A_x_) × 100/A_0_, where A_0_ was the absorbance of the control (~0.800, ABTS solution with water), and A_x_ was the absorbance of the sample. The antioxidant potential was expressed as a percentage of ABTS radical inhibition. Ascorbic acid at the concentration of 0.2 mg/mL was used as a control substance.

#### 4.7.2. DPPH Assays

The absorbance of DPPH radicals (2 mL) and the samples (20 µL) was measured at 517 nm, and the scavenging capacity of the samples was calculated as % of inhibition = (A_0_ − A_x_) × 100/A_0_, where A_0_ was the absorbance of the control (~0.800, DPPH solution with water), and A_x_ was the absorbance of the sample. The antioxidant potential was expressed as a percentage of DPPH radical neutralization. Ascorbic acid (0.2 mg/mL) was used as a control.

All absorbance readings are performed on a UV spectrophotometer, UV-1800 (Shimadzu, Kyoto, Japan).

### 4.8. Size, PDI, Zeta Potential, Conductivity, and Mobility Analyses

The size, PDI, zeta potential, conductivity, and mobility of empty and rosehip extract-loaded liposomes (native and UV-irradiated) were determined via photon correlation spectroscopy (PCS) in Zetasizer Nano Series, Nano ZS (Malvern Instruments Ltd., Worcestershire, UK). Each sample was 500-fold diluted and measured three times at room temperature.

### 4.9. UV-Stability Study

In order to examine liposomes stability after exposure to UV irradiation, empty and extract-loaded liposomes (3 mL) were exposed to UV-C irradiation (253.7 nm) in a quartz tube at room temperature during 20 min [14,68,69]; subsequently, TPC, antioxidant capacity, size, PDI, zeta potential, conductivity, mobility, DSC, and Raman analyses were performed.

### 4.10. Raman Spectroscopy

The Raman spectra of pure rosehip extract, Phospholipon (a commercial mixture of phospholipids), empty liposomes, rosehip extract-loaded liposomes, as well as the UV irradiation exposed counterparts, were collected using an XploRA Raman spectrometer (Horiba Jobin Yvon, Palaiseau, France) equipped with microscope Olympus BX51 in the spectral range from 250 to 3500 cm^−1^. Raman scattering was excited by a laser at a wavelength of 532 nm equipped with 1200 lines mm^−1^ grating, spectra were recorded by applying 120 acquisition time, using a 100% filter. Autocalibration was performed using the 520.47 cm^−1^ Raman frequency shift in silicon. In order to assess a possible sample inhomogeneity, ten spectra were recorded for each investigated sample. The spectra pre-processing was realized using Spectrograph software [70].

### 4.11. DSC Analysis

DSC is the most frequent technique that is used for the determination of the phase transition and enthalpy changes of model lipid membranes and phospholipid bilayer as a carrier for various active compounds [32]. Thus, the thermal properties of pure Phospholipon, lyophilized rosehip extract, and empty and extract-loaded liposomes were analyzed in a DSC60Plus differential scanning calorimeter (Shimadzu, Kyoto, Japan). The samples were placed in hermetic aluminum pans (4 ± 0.5 mg) under a nitrogen purge gas flow of 50 mL/min. The phase transition changes were monitored from 0 to 350 °C, with a heating rate of 10 °C/min.

### 4.12. Statistical Analysis

In the present study, the statistical analysis was performed by using analysis of variance (one-way ANOVA) followed by Duncan’s *post hoc* test within the statistical software, STATISTICA 7.0. The differences were considered statistically significant at *p* < 0.05, n = 3.

## 5. Conclusions

This work presented the encapsulation of rosehip extract, rich in various bioactive compounds, into liposomes using the simple proliposome method. The encapsulation efficacy was high, justifying the encapsulation procedure. The characterization of both non-irradiated and UV-irradiated liposomes showed that UV irradiation did not change the main physical properties of extract-loaded liposomes, as well as their antioxidant ability. However, UV irradiation caused a decrease in the content of isoquercetin, rutin, hyperoside, quercitrin, and chlorogenic acid in extract-loaded liposomes. A slight difference was detected in the DSC spectra of UV-treated liposomes, which implies the potential degradation of carotenoids during irradiation. Raman spectroscopy indicated that the extract was completely covered by the bilayer lipid membrane during liposome entrapment and that the peroxidation process was minimized by the presence of rosehip extract loaded into liposomes. These results may be a guide to the potential application of liposomes in foods, pharmaceuticals, or cosmetic industries, especially when treatment of liposomes using UV irradiation is needed, e.g., in the sterilization procedure.

## Figures and Tables

**Table 1:** Size, polydispersity index (PDI), zeta potential (ζ), conductivity (G), and mobility (µ) of empty liposomes and rosehip extract-loaded liposomes and encapsulation efficiency (EE), before and after UV irradiation.

	L	L_UV_	L + e	L + e_UV_
**EE [%]**	n.a. ^1^	n.a.	90.8 ± 0.4 ^a^	91.0 ± 0.8 ^a^
**size [nm]**	363.2 ± 7.8 ^b^	361.0 ± 4.2 ^b^	251.5 ± 7.2 ^a^	247.3 ± 5.6 ^a^
**PDI**	0.376 ± 0.023 ^a^	0.387 ± 0.010 ^a^	0.439 ± 0.027 ^b^	0.428 ± 0.003 ^b^
**ζ [mV]**	−21.7 ± 0.7 ^a^	−21.3 ± 0.5 ^a^	−22.2 ± 0.5 ^a^	−22.4 ± 0.7 ^a^
***G* [mS/cm]**	0.004 ± 0.000 ^b^	0.004 ± 0.000 ^b^	0.007 ± 0.001 ^a^	0.008 ± 0.001 ^a^
***µ* [µmcm/Vs]**	−2.66 ± 0.10 ^b^	−2.51 ± 0.09 ^b^	−1.92 ± 0.15 ^a^	−1.96 ± 0.05 ^a^

^1^ Values with the same letter (a, b) in each row showed no statistically significant difference (*p* > 0.05; n = 3; analysis of variance, Duncan’s *post hoc* test; mean value ± standard deviation); L, liposomes; e, extract; n.a., not applicable.

**Table 2:** The content of individual compounds in *Rosa canina* extract and extract-loaded liposomes (non-treated and UV-irradiated liposomes) determined in HPLC analysis.

Sample	Chlorogenic Acid	Rutin	Hyperoside	Isoquercetin	Quercitrin
	µg/mL				
Extract	45.02 ± 0.87 *	159.12 ± 1.13	81.46 ± 1.04	185.57 ± 1.17	77.23 ± 1.25
	µg/g				
Extract-loaded liposomes	46.03 ± 0.54	169.29 ± 1.40	28.79 ± 0.28	355.27 ± 2.15	4.45 ± 0.12
Extract-loaded liposomes_UV_	43.68 ± 0.98	152.32 ± 1.22	21.44 ± 0.84	329.18 ± 1.94	*tr.*

* The results represent the means of three determinations ± standard deviation (SD); *tr.* traces.

**Table 3:** Thermodynamic profiles of pure Phospholipon, pure rosehip extract (E), unloaded and extract-loaded liposomes (L, UV-irradiated and non-treated samples); peak temperatures and enthalpy changes (ΔH).

Sample	Temperature (°C)			ΔH (J/g)
	Onset	Peak	Offset	
Phospholipon	121.4	138.7	159.4	−22.7
	225.2	233.6	241.0	−2.9
	306.6	333.9	347.3	−31.6
E	79.8	87.8	115.9	−38.5
	165.6	174.4	181.3	−7.5
E UV	81.9	89.5	100.4	−8.9
	164.8	174.5	182.9	−2.2
L	140.9	152.4	177.7	−30.5
	220.25	232.1	239.9	−4.2
L UV	141.4	168.4	199.5	−20.9
	223.73	232.1	237.9	−1.61
L + e	113.8	125.6	155.5	−32.8
	248.3	260.3	292.2	−24.81
L + e UV	119.2	127.6	133.3	−8.9
	246.9	262.3	293.5	−5.4

## Data Availability

The datasets generated during and/or analyzed during the current study are available from the corresponding author on reasonable request.
